# One-year follow-up of a sit-stand workstation intervention to decrease sedentary time in office workers

**DOI:** 10.1016/j.pmedr.2019.01.008

**Published:** 2019-01-16

**Authors:** Nirjhar Dutta, Thomas Walton, Mark A. Pereira

**Affiliations:** a Department of Medicine, University of Minnesota, Minneapolis, MN, United States of America; b Department of Anthropology, University of Minnesota, Minneapolis, MN, United States of America; c Division of Epidemiology & Community Health, School of Public Health, University of Minnesota, Minneapolis, MN, United States of America

**Keywords:** Sit stand desk, Workstation, Sedentary time, Sitting, Standing, Light physical activity, Productivity, Focus group, Qualitative methods

## Abstract

**Background:**

Prolonged sedentary time is associated with adverse health outcomes, after controlling for the role of moderate-to-vigorous physical activity. We previously reported on a four-week randomized trial using a sit-stand desk (SSD) intervention that decreased sedentary time at work without changing activity level during non-work hours.

**Purpose:**

The purpose of this study was to measure the impact of the SSD on sitting time and activity level one year after the original intervention.

**Methods:**

A pre-post design was used where the control period from the original study was regarded as “pre” and the measurements made in the follow-up study as “post.” The follow-up study was conducted in the same office workers over a two-week period in June 2013.

**Results:**

Fifteen out of the 23 participants took part in the follow-up study. Self-reported sitting time during work-hours was decreased by 22% (95% CI: 15% to 29%; *p* < 0.001), replaced almost entirely by standing. Activity measured by Gruve accelerometer during work-hours were significantly higher in the one-year follow-up period compared to baseline (+24,748 AU/h; 95% CI: 7150 to 42,347; *p* < 0.01). Sedentary time during work-hours was decreased by 0.77 min per work-hour (95% CI: −1.88 to 0.33 min/h; *p* = 0.17). Qualitative findings through focus group sessions suggested the workers had overall favorable experiences with the SSDs without negatively impacting productivity.

**Conclusion:**

One year following the original intervention, participants continue to have increased activity and decreased sedentary time at work with the use of SSDs.

## 1 Introduction

Prolonged sedentary time is associated with adverse health outcomes, after controlling for moderate-to-vigorous physical activity, with the possible exception of extreme levels of PA (>35 METS per week) (Bankoski et al., 2011; Ekelund et al., 2016; Finni et al., 2014; Hamilton et al., 2007; Owen et al., 2010). We have previously reported a randomized cross-over study evaluating use of adjustable sit-stand desk (SSD) in the workplace to reduce sitting time and replace sedentary activity with light (non-exercise) activity (Dutta et al., 2014). That intervention reduced sitting time at work between 21% (by accelerometer) to 40% (by survey) and replaced sedentary time with light activity by ~5 min per hour at work (by accelerometer); which amounted to sedentary time being reduced by about 40 min per workday. We also reported, from focus group and interviews, that participants were highly satisfied with the SSDs, experienced greater energy/alertness and had increased face-to-face interaction with co-workers without hampering productivity or privacy (Dutta et al., 2015).

There have been many studies aimed at reducing sitting time at work. A 2016 meta-analysis of such workplace interventions showed that in the short term (up to 3 months) the impact of SSDs on reducing sitting time with standing is about 30 min to 2 h per workday (Shrestha et al., 2016). However, most studies evaluated the interventions in the short term, with only a few examining longer-term effects; two such studies found a 45–53 min decease in workplace sitting at the 12–18 months follow-up (Healy et al., 2016; Zhu et al., 2018).

The purpose of the current study was to conduct a one-year follow-up from the end of the original study to determine, via quantitative and qualitative methodology, whether the effects of SSDs continued a year later. Two hypotheses were tested in this study: 1) participants would have less sitting time and more standing time compared to their control period in the original study; 2) participants would have higher overall PA and less sedentary time during work as compared to their control period in the original study.

## 2 Methods

The study was approved by the University of Minnesota's institutional review board and was registered on clinicaltrials.gov (NCT01863056).

In this 1-year follow-up from the end of the original study, a pre-post design was used where the control period from the original study was regarded as “pre” and the measurements made in the follow-up study was regarded as “post.” The follow-up study was conducted in the same group of office workers at the same company in the Twin Cities Metro Area, MN, USA (*Caldrea*, Inc.) over a two-week period in June 2013.

Eligible participants were current employees of the company who completed the original study, regardless of whether they still had their SSD and how much they use it currently. Those with significant musculoskeletal problems or varicose veins as well as pregnant women were excluded. Two months before the start of the follow-up study, an email was sent to all the eligible participants explaining the study and informed consent form. Demographic information, work schedule, pertinent health history of the participants were obtained during an in-person consultation with investigators prior to start; at that visit, written informed consent was collected and study-related equipment (e.g. accelerometer) were distributed.

The intervention in this follow-up study was the SSDs (*Ergotron* Inc., Eagan, Minnesota, USA), which allowed users to switch between sitting and standing by pushing on a mechanical lever. These desks were left with the participants at the end of the original study, in April 2012. For the two weeks of the follow-up study, participants were explicitly asked not to change their normal usage of the SSDs because the goal was to determine their regular (“free-living”) usage pattern. Hours spent at work were determined from participant's self-reported schedule.

Sitting and standing time was assessed by the validated Occupational Sitting and Physical Activity Questionnaire (OSPAQ) (Chau et al., 2012). The survey was loaded onto a study specific survey website hosted by *SurveyMonkey.com* (USA) and a weblink was emailed to the participants at the end of each week. The findings of the survey were averaged for each period.

PA was measured via the same validated accelerometer (*Gruve ®, Muve* Inc. Minneapolis, MN) used in the original study that participants wore on the hip during all waking hours (4 weeks during the baseline period and 2 weeks during the follow up) (Manohar et al., 2012). Data were analyzed in activity units per hour (AU/h), the raw data from the Gruve accelerometer. The AU/h was converted to the four aspects of activity: Sedentary (0–1 metabolic equivalent of task), light (1–3 MET), moderate (3–6 MET), and intensive (6+ MET) (Dutta et al., 2014; Levine et al., 2012)**.**

.Qualitative data were collected using focus group methods. At the end of the two week follow-up period, researchers conducted two 60-minute focus groups in a private conference room: one with supervisory staff and another with non-supervisory staff. The interviews were recorded and transcribed. Focus group interviews revisited the same topics discussed during the initial study and focused on perceptions of SSD impact on workplace productivity and health over time (Dutta et al., 2015). The data analysis approach was derived from grounded theory: first, an open-coding phase was used to identify initial themes, then categories and properties associated with these themes were clarified in a secondary axial coding stage (Charmaz, 2006; Strauss and Corbin, 1990). Codes were applied separately by researchers in order to increase reliability and then any interpretive differences were discussed and resolved.

Mixed-effects linear regression for repeated measures was used to analyze data with STATA (‘*xtmixed*’ *STATA* 11, College Station, Texas). Adjustment for other covariates, such as age, sex, or body mass index was not necessary because each person serves as his/her own control. A type I error of α < 0.05 was accepted as statistically significant, and 95% confidence intervals were computed.

## 3 Results

Twenty-eight participants were eligible for the follow-up study. Five participants were no longer with the company, leaving 23 available. One did not participate due to time constraints, one did not participate due to loss of interest, four did not participate for unknown reasons, one met exclusion criteria due to pregnancy, and one met exclusion criteria due to lack of availability for data collection. Therefore, 15 participants participated in the follow-up study (15/23 = 65% participation rate). All of them were still using the SSDs. Participants were on average 39.1 (sd = 9.7) years old; 11 were female, four were male; were of average body weight (mean BMI = 25.2, sd = 5.0); were mostly full-time workers (mean work time 37.5 h/wk., sd = 4.8). Baseline characteristics of the follow-up study participants were very similar to those of the original total study sample (i.e, original sample means: age = 40.6, BMI = 25.6).

**Fig. 1:**
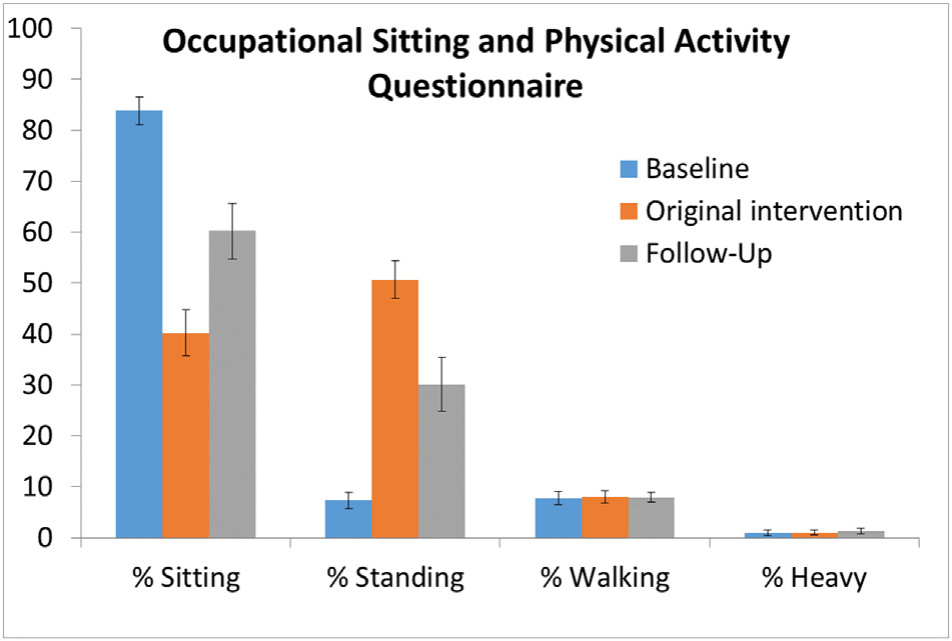
Sitting, standing, walking, and heavy activity from the OSPAQ survey at the original study baseline and 1-month, as well as during the 1-year follow-up (mean, standard error; *n* = 14).

First primary outcome regarding sitting time at work was evaluated with the OSPAQ survey. There were 61 completed OSPAQ surveys by participants (out of possible 90). They reported reducing sitting time by 22% (95% CI: 15% to 29%; *p* < 0.001) during the 1-year follow-up as compared to baseline control period. On average, the decreased sitting time was replaced with standing as walking and heavy work was not different between control and follow-up (*p* = 0.77 and *p* = 0.29 respectively). Fig. 1 shows OSPAQ survey results for the original study (baseline) and the follow-up study and compares it to results from the original study.

Second primary outcome regarding overall activity and sedentary vs. non-sedentary time at work was evaluated with the Gruve accelerometer. The accelerometer data are based on 5562 h, out of which 2873 h were recording during work-hours across all three time points (baseline, original study, and 12-month follow-up). Results are shown in Table 1. For overall activity, compared to baseline, AU per work hour was significantly higher after 1-month of the original intervention as well as at the 12-month follow-up. AU/h for total hours and non-work hours were not statistically different between control and intervention period (*p* = 0.51 and *p* = 0.98 respectively; data not shown). For sedentary time at work, accelerometer data demonstrated 24.9 min/h in a sedentary state during the original baseline of the trial, which was reduced to 19.2 min/h at the end of the original 1-month intervention (*p* < 0.01). During the 12-month follow-up, mean sedentary time at work was 24.1 min/h, a reduction of sedentary time of 0.77 min per hour (95% CI: -1.89 to 0.32 min/h; *p* = 0.17) (Table 1).

**Table 1:** Activity unit per work hour and sedentary time per work hour at baseline, and treatment effects at one-month and 12-month follow-up (*n* = 15).

	Baseline (Mean, SE)	Treatment effect in the original study	Treatment effect at12 month follow-up
Activity unit per work hour	205,272 ±12,967	+26,070 (95% CI: 11606 to 40,534; *P* < 0.01)	+24,748 (95% CI: 7150 to 42,347; P < 0.01)
Sedentary time (min) per work hour	24.9 ±1.46	−5.4 (95% CI: −6.66 to −4.99; P < 0.01)	−0.78 (95% CI: −1.88 to 0.33; *P* = 0.17)

Secondary outcome regarding participants' experience with SSDs was evaluated with focus group sessions. Focus group data indicate that participants continued to regularly use SSDs and experienced improved energy, as well as increased postural awareness. Accessories to aid in standing, such as anti-fatigue mats, foot-stool, and comfortable shoes, were perceived as helpful but were underutilized because of the effort required. The participants did not perceive normal workflow to be negatively impacted. While participants found that SSDs sometimes limited workspace, they did not experience a negative impact on productivity; many noted that they liked having the ability to divide tasks into “sitting” and “standing” depending on the nature of the effort involved. Interpersonal communication was sometimes enhanced as participants found that SSDs allowed them to share monitors more and pose ad-hoc questions to colleagues more easily. SSDs did not cause participants to experience a decrease in privacy.

## 4 Discussion

It is important to determine whether SSDs will continue to be used, and influence activity levels, well past the originally planned trial period.

Our findings are based on valid subjective and objective measures of sitting time and PA. During the one-year follow-up, subjective reports based on the OSPAQ indicated 22% absolute reduction in sitting at work relative to the baseline period of the original trial; which translates to a 105 min reduction of sitting time for each 8-h workday. For comparison, the acute effect of the original 1-month intervention was a 40% absolute reduction in sitting at work. In the original study, sitting and standing time was measured objectively with an accelerometer as well as via the OSPAQ survey, and objective sitting time was 50% more and objective standing time was 50% less than the subjective times (Dutta et al., 2014). Taking into account this discrepancy, the true reduction in sitting time may be closer to 52 min per 8 h workday, which is similar to findings in other studies. In one study, participants decreased sitting time by 53 min in an 8 h workday using SSDs (Zhu et al., 2018). Another study found that participants decreased sitting time by 45 min in an 8 h workday at 12 month (Healy et al., 2016).

The subjective assessment results were somewhat corroborated by the objective accelerometer findings. Overall PA during the work day remained ~12% higher during the 1-year follow-up relative to baseline. However, the finding for objectively measured sedentary time during the 1-year follow-up was small, 0.77 min/h, translating to 30 min of sedentary time being replaced with non-sedentary time for a 40-h work week. In the original study, participants reduced sedentary time during work-hours by 4.8 min/h. At the 12 month follow-up, overall activity level of the participants were still higher than at baseline (similar to original intervention); however, sedentary time at work was not as low as it was in the original intervention period. Interestingly, participants were spending more time in vigorous PA at work (1.63 min/workhour at baseline vs. 2.21 min/workhour at follow-up, *P* < 0.01). We suspect that longer-term and more sustained worksite behavioral and environmental interventions will be needed to foster more sustained behavioral impacts on sedentary time and overall physical activity (Buman et al., 2017; Healy et al., 2016; Healy et al., 2017).

The qualitative findings indicate that workers had overall favorable experiences with the SSDs and did not perceive normal workflow to be negatively impacted (e.g. productivity, interaction with co-workers, privacy concerns) (Dutta et al., 2015). Thus, this study appears to have generalizability to similar office-based sedentary workers.

This follow-up study has limitations. First, like most lifestyle interventions, it could not be blinded and subjective measures were used for sitting time. However, activity was objectively measured with the Gruve accelerometers, which should minimize bias. Sample size was small and a pre-post design was used. However, using each person as his/her own control is a major methodological strength and improves statistical power.

In conclusion, this study adds to the few studies that have looked at long-term follow-up of an intervention that focused solely on use of SSDs in full-time working sedentary adults. The findings suggest that use of SSDs continue to impact sitting time and sedentary time one year after installation.
